# Synergetic Effects of Graphene Nanoplatelets/Tapioca Starch on Water-Based Drilling Muds: Enhancements in Rheological and Filtration Characteristics

**DOI:** 10.3390/polym13162655

**Published:** 2021-08-10

**Authors:** Maqsood Ahmad, Imtiaz Ali, Muhammad Syahmi Bins Safri, Mohammad Arif Izzuddin Bin Mohammad Faiz, Asif Zamir

**Affiliations:** Department of Petroleum Engineering, Universiti Teknologi PETRONAS, Seri Iskandar 32610, Malaysia; muhammad_24433@utp.edu.my (M.S.B.S.); mohammad.arif_24250@utp.edu.my (M.A.I.B.M.F.); asif.zamir@utp.edu.my (A.Z.)

**Keywords:** graphene nanoplatelets, mud rheology, filtration properties of water-based muds, tapioca starch rheology, filtercake morphology

## Abstract

Several borehole problems are encountered during drilling a well due to improper mud design. These problems are directly associated with the rheological and filtration properties of the fluid used during drilling. Thus, it is important to investigate the mud rheological and filtration characteristics of water-based drilling muds (WBMs). Several materials have been examined but due to the higher temperature conditions of wells, such materials have degraded and lost their primary functions. In this research, an attempt was made to prepare a water-based mud by utilizing graphene nano platelets (GNP) in addition to the native tapioca starch at different ratios. The combined effect of starch and graphene nano platelets has been investigated in terms of mud’s rheological and filtration parameters, including its plastic viscosity (PV), yield point (YP), fluid loss volume (FLV) and filtercake thickness (FCT). The morphological changes in the filtercake have also been observed using Field Emission Scanning Electron Microscope (FESEM) micrographs. Plastic viscosity was increased from 18–35 cP, 22–31 cP and 21–28 cP for 68 °F, 250 °F and 300 °F, respectively. The yield point was also enhanced from 22–37 lb/100ft^2^, 26–41 lb/100ft^2^ and 24–31 lb/100ft^2^ at the studied range. The fluid loss was dramatically reduced from 14.5–6.5 mL, 17.3–7.5 mL and 36–9.5 mL at 68 °F, 250 °F and 300 °F respectively. Similarly, filtercake thickness was also reduced which was further illustrated by filtercake morphology.

## 1. Introduction

The exploration and production (E&P) industry is looking for new resources in unexplored regions and deeper formations to cater to the increasing demand for oil and gas. The success of any drilling operation is dependent on the proper design of the drilling mud used. It is a circulating fluid used in rotary drilling to deliver various functions. The mud properties affect the efficiency of the drilling operation and should handle the bottom hole high pressure high temperature (HPHT) conditions. Moreover, most of the drilling problems are mainly related to the inappropriate selection and inadequate design of the drilling fluid [1,2,3,4,5,6].

Rheological and filtration characteristics of water-based muds are very important to maintain during drilling operations. For optimal mud properties, several materials including bentonite, polymers, salts, inhibitors, and weighting agents are added into mud for improving its properties. Bentonite is added to drilling fluids for improving the rheological properties and reducing the filtrate loss by forming a filtercake with reduced permeability. The majority of high-quality bentonite is composed of montmorillonite, with minor amounts of other minerals. In general, bentonites contain some amounts of other clay minerals such as elites, kaolinites, and chlorites, as well as non-clay components including quartz and feldspar etc. Because montmorillonitic clays have the greatest swelling capacity (inducing viscosity and the formation of low permeability filter cakes), the presence of other components in significant amounts can degrade the quality of the bentonite. In addition, owing to its high specific surface area, cation exchange capacity, and hydration capabilities, bentonite is a desirable drilling fluid agent. The key to achieving appropriate rheological and filtration characteristics for muds subjected to high temperatures is to maintain the bentonite dispersion, which requires the use of suitable dispersants. In the high temperature conditions of a well, the flocculation of bentonite particles allows particles to fuse together to establish a loose and permeable network, which boosts filtrate losses and impacts bentonite performance. Hence, some thermally stable polymers or other additives are added into the mud system to keep the particles dispersed in the solution.

Starch is the second most abundant biomass found in nature [7,8], with almost no negative effects on the environment [9]. Starch and natural gums were first used in 1937 as a biopolymer for controlling fluid loss volume in bentonite mud. Until now, starches are widely used in drilling fluid applications because of their availability, biodegradability, versatility, renewability, and low cost among various polymers [7,9,10,11,12]. Various starches have been used to modify the properties of mud and have been found to be a very effective and environmentally friendly alternative as compared to commercially available additives. Talukdar, et al. [13] experimentally tested two naturally available starches, including banana and corn starch, at various concentrations. Both starch types were discovered to be excellent fluid loss control agents in a non-damaging drilling fluid. Nyeche, et al. [14] reported in a study that the blend of PAC and potato starch in equal percentages is the appropriate additive for the improvement of the fluid loss control properties of drilling mud. Better thermal stability of mud has also been observed for this combination with a minimal increase in viscosity. Another study used cassava starch to improve viscosity and control fluid loss volume in bentonite-based mud. Owing to the swelling ability of the starch flour, the viscosity and suspension capability of mud was increased with an increase in concentration at 80 °C. Cassava starch with a concentration of 2 g showed the lowest fluid loss volume and a reduction of about 8% in filtrate volume within the studied range [15].

Nanotechnology has the ability to improve drilling fluid performance for drilling in deeper and harsher environments. Nanoparticles exhibit some special properties, such as their small size (1–100 nm), high specific surface area, optical, thermal, electrical, mechanical and excellent adsorption ability. Because of the small quantity of nanoparticles, it is economical for any industrial application. In drilling fluid, nanoparticles were effectively evaluated to control mud rheology, prevent mud invasion into the formation, and generate a thin and compact filtercake at HPHT conditions [16]. Graphene is a single layer of graphite with unique features, and it has recently been the focus of numerous studies. It is a two-dimensional planar sheet of sp2-bonded carbon atoms with exceptional mechanical, electrical, thermal, and physical characteristics. It has been extensively used in a variety of applications due to its intriguing features [17,18,19,20,21,22]. Numerous researchers examined the significance of various metallic and non-metallic nanoparticles in WBMs. For example, Ismail, et al. [23] experimentally accessed the usage of multiwalled carbon nanotubes (MWCNT) and graphene nano platelets to reduce the coefficient of friction and fluid loss volume of water-based drilling mud. In addition, the rheology was also determined. The results showed that the addition of MWCNT minimized the torque lubricity between 38–59%. A marginal enhancement in mud rheological properties and filtration properties were also observed. In another study, Parizad, et al. [24] reported a polymeric water-based drilling fluid containing TiO_2_ nanoparticles to enhance mud’s rheological, thermal, and electrical conductivities of the fluids. The results demonstrated that the addition of TiO_2_ particles not only enhanced the rheological behavior but also improved the thermal and electrical conductivities of the drilling fluids up to 25% and 41%, respectively. The filtrate volume was decreased up to 27%. Experimentally we studied the rheological and filtration properties in terms of plastic viscosity, yield point and fluid loss volume [25]. It was reported that the rheological properties were enhanced using graphene nano platelets (GNPs) in the mud blends. Moreover, the fluid loss volume (American Petroleum Institute (API) and HPHT) was also reduced with the addition of GNPs. By comparing GNP with other nanomaterials, it was observed that GNP has better performance than MWCNT and nano silica (NS) at the same concentration in the studied parameters. Taha and Lee [26] evaluated graphene in WBM and reported that it improved the rheological and filtration performance of the drilling fluids. The drilling’s rate of penetration (ROP) was enhanced while the filtrate loss and torque were minimized. Other studies related to the addition of GNP in drilling fluids also showed significant improvements in the mud properties [26,27,28]. Despite all of GNP’s merits in WBMs, there are some challenges when using it in muds. One of most crucial shortcomings is its poor dispersion characteristics in WBMs. In order to address this issue, various additives have been tested and reported in the latest literature [19,21].

The given discussion motivates researchers to investigate new potential agents that can perform better in HPHT environments. In this study, a hybrid additive including varied compositions of GNPs and tapioca starch was introduced to the base fluid. The applicability and versatility of the new material as a potential additive for enhancing rheological properties and lowering fluid loss in different muds are discussed in detail. In addition, a study of rheological stability under high temperature conditions was performed to examine the stability of the proposed solution under static conditions.

## 2. Materials and Methods

### 2.1. Materials

In the current work, bentonite and graphene nano platelets (GNP: Purity > 95%) were purchased from Sigma-Aldrich. Barite and polyanionic cellulose (PAC-R) were obtained from Scomi Oiltools Sdn. Bhd. Tapioca starch was purchased from a local enterprise in Malaysia. For pH control, potassium hydroxide (KOH) was added and was procured from R&M chemicals. Deionized water was utilized as a base fluid in all mud samples. All chemicals were reagent grade and were used as received without further purification.

### 2.2. Chemical Composition and Characterization of Bentonite

Table 1 summarizes the chemical composition of natural sodium bentonite. The major components of bentonite were SiO_2_, Al_2_O_3_, Na_2_O, MgO, CaO, TiO_2_ and Fe_2_O_3_. The concentrations of CaO and Na_2_O (K_2_O) in montmorillonite represent interlayer cations. The amount of CaO and Na_2_O in a bentonite determines whether it is Ca-bentonite or Na-bentonite.

In the studied bentonite sample, the total content of SiO_2_ and Al_2_O_3_ is 81.58%, and the corresponding ratio of SiO_2_ to Al_2_O_3_ is 4.29, which indicates a higher SiO_2_ content as compared to others. On the other hand, the sodium content is higher than calcium content, therefore, this kind of sample belongs to the sodium bentonite category.

X-ray diffraction (XRD, PAnalytical Xpert Powder) was used to determine the mineralogical composition of bentonite and graphene nano platelets. It is a rapid analytical technique mainly used to identify phases in a crystalline material. For XRD measurements, approximately 2 g of powder was placed in an acrylic sample holder with a depth of 3 mm. Parallel beam optics at 40 kV and 40 mA were used to examine the sample. The sample was scanned for reflections (2θ) in intervals of 0.02° with a 2 s count time per step from 5° to 85°. The resulting peaks were analyzed using X’Pert HighScore Plus software. Additionally, the morphological structure of GNP was characterized using transmission electron microscopy (TEM).

### 2.3. Preparation of Mud Samples

For the preparation of water-based mud samples, the API standard laboratory protocols were followed. GNPs were dispersed in deionized water using ultrasonication before being added to the mud samples to reduce the aggregation tendencies. This ultrasonication helped the mud system’s colloidal stability. A previous method by Ponmani, et al. [29] was followed with some modification. Briefly, a beaker was taken with a measured quantity of GNPs in the deionized water and sonication was carried out at 25 KHz and 450 W for one hour. A laboratory barrel, equivalent to 350 mL of base mud was prepared by adding deionized water, bentonite, polyanionic cellulose (PAC-R), tapioca starch, GNP and barite in order. These additives were first mixed properly using a Fann multimixer at 11,500 rpm to ensure thorough dispersion of all additives and to confirm that no lumps were formed because this may affect the nanofluid stability. The formulation of the studied WBMs is detailed in Table 2 and Table 3.

### 2.4. Determination of Mud Density

The mud density was determined following API recommended practices. A Fann mud balance instrument with an accuracy of 0.01 g·cm^−3^ was used to calculate the mass per volume of a drilling fluid.

### 2.5. Measurement of Rheological Properties

Any fluid’s resistance to flowing is referred to as rheology. It is an important feature of every drilling fluid and must be properly designed [30,31]. The rheological properties of bentonite-based drilling mud in terms of plastic viscosity (PV) and yield point (YP) were assessed at ambient and hot roll temperature (250 °F and 300 °F) conditions. These properties were accessed to investigate the impact of graphene nano platelets in combination with starch. All the PV and YP tests were carried out according to the API specifications. The mud samples were prepared as described in a previous section, and the amount of the GNP was added to the blends varied in the range 0–1.5 g. The rheological properties of the formulated mud samples were measured using a Fann 35 A viscometer. The viscometer used in this work gives dial readings at 600, 300, 200, 100, 6 and 3 rpm. For PV and YP measurements, the dial readings of 600 and 300 rpm were considered by using Bingham plastic equations,

(1) $$\mathrm{PV}=R_{600}-R_{300}$$

(2) $$\mathrm{YP}=R_{300}-\mathrm{PV}$$

where R_600_ and R_300_ are the dial readings at 600 and 300, respectively. 

### 2.6. Measurement of Filtration Properties

The filtration rate of a properly designed drilling mud should be minimal, especially when dealing with HPHT conditions. Drilling fluid may degrade under high temperatures, and the mud may have an excessive filtrate intrusion, resulting in a loss of resources and money. Thus, the drilling engineer should design a drilling fluid with the best possible specifications [32,33]. Any mud must undergo filtration testing to determine fluid loss and filter-cake thickness. The quantity of particles in the drilling mud, temperature, pressure, as well as the physical and chemical reactions within it, are the controlling factors of these parameters. In the current work, both API and HPHT filtration tests were performed. For API filtration, the API filter press (Fann Instrument Company, Houston, TX, USA) was used and pressure was maintained at a fixed pressure of 100 psi at room temperature conditions for 30 min as recommended by API standards. To mimic the static filtration behavior of drilling mud, the HTHP static filtration tests were carried out using the Ofite HTHP filterpress. Similarly, in HPHT tests, the pressure was kept constant at 500 psi, and the temperature varied from 250 °F and 300 °F. After each run, the filtercake was carefully stored after removing the access mud. Finally, the filtercake thickness was measured with a digital vernier caliper.

### 2.7. Thermal Ageing

Drilling muds are often exposed to high pressure and high temperature conditions in deep wells. Thus, the mud properties can fluctuate after being exposed to such environments for a longer duration, and the drilling fluid would no longer have the same characteristics as before. As a result, monitoring and analyzing such changes are important in drilling fluid analysis. Hot rolling (dynamic) at high temperatures could be used to mimic this process in the lab, allowing researchers to observe the variations in the mud properties. The thermal stability of all the formulated mud samples was evaluated by ageing them for 16 h at 250 °F and 300 °F using Fann hot roll oven (Fann Instrument Company, USA). Moreover, the mud was also agitated using the specially designed power-driven rollers. After ageing, the rheological characteristics of the drilling fluids were measured using the API standard.

## 3. Results and Discussion

### 3.1. X-ray Diffraction (XRD) of Bentonite and Graphene Nano Platelets (GNP)

**Figure 1:**
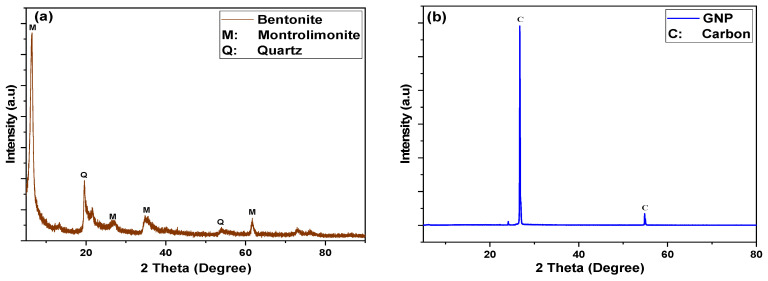
X-ray diffraction (XRD) spectrum of (**a**) bentonite and (**b**) graphene nano platelets (GNP).

Figure 1a shows the XRD pattern of the studied sample of bentonite. The material is predominantly composed of montmorillonite, as shown by the distinctive features at *d*_001_ was 12.27 Å reveals a sodium preponderance, allowing the samples to be classified mostly as sodium bentonite (Na-bentonite). The bentonite clay is majorly composed of montmorillonite with some amounts of quartz and other minerals present in varying amounts as observed from the XRD data. The montmorillonite has peaks with 2θ at 7.15, 26.44, 34.94 and 61.73° while quartz showed peaks at 20.82 and 40.14°. Similar patterns have also been observed by other researchers [34,35,36,37,38]. These minerals, in small portions, are thought to have a slight effect on the mud’s dispersion characteristics. The major constituents, which account for nearly 90% of the total weight, are silica, aluminum, magnesium and iron.

Figure 1b displays the X-ray diffraction pattern of GNPs which revealed intensive peaks at 2θ at 26.54° and 54.66° corresponding to the planes (002) and (004) of carbon, which strongly validates graphene. The first peak at 26.54° showed a full width at half maximum (FWHM) of 0.56°.

### 3.2. Morphology of GNP

**Figure 2:**
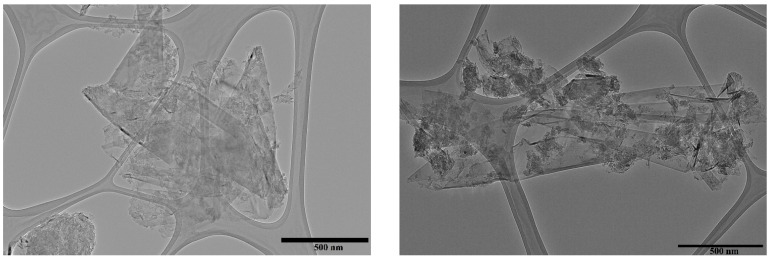
Transmission electron microscopy (TEM) micrographs of GNP used in drilling mud.

TEM images were obtained to compare XRD information in order to confirm the thickness of graphene nanoplatelets. Figure 2a,b shows representative images which demonstrate that a clear view of the GNP is described by the TEM analysis. It was determined that the thickness of the micrographs was generally less than 10 nm, that is inferior to the crystal domain computed from XRD measurements and is in agreement with the manufacturer’s data sheet. GNP exhibited a typical morphology, with finely dispersed graphene nanosheets extending over a few microns in the lateral dimensions. Small overlapping sections revealed the existence of several smaller GNP sheets as well. It is worth mentioning that GNP sheets are wrinkle-free, demonstrating that there are no other elements on their surface. 

### 3.3. Density of Mud

The mud weight of any fluid is an important property to be considered while designing a mud for overbalanced drilling. To ensure optimal penetration rate in a drilling process, mud weight is designed slightly higher than formation pressure. A high mud weight may result in borehole problems such as loss of circulation, formation damage, pipe sticking, and a reduction in penetration rate. While an inadequate mud weight may also induce well collapse or instability issue because of the significant difference between formation and hydrostatic pressure. Therefore, selecting an optimal mud weight during the mud design stage is essential to achieve a successful drilling operation while being cost-effective. 

**Figure 3:**
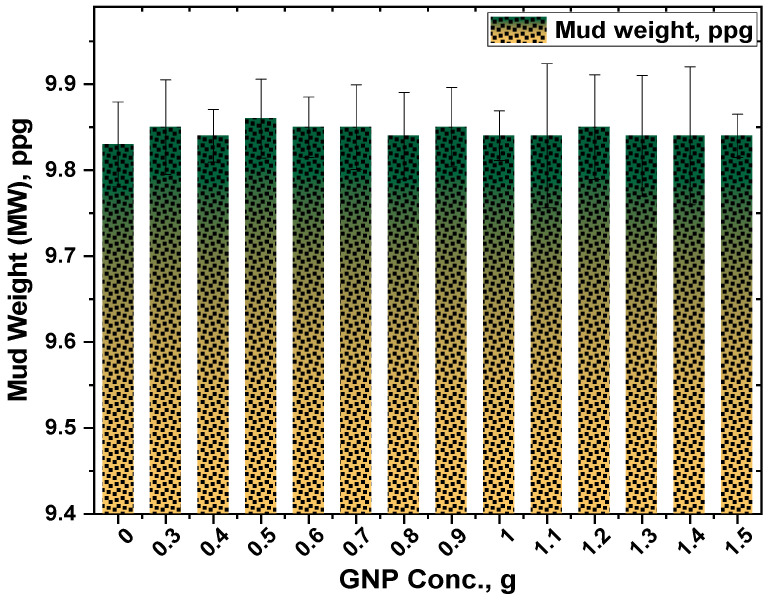
Response of GNP-Starch on the density of the formulated muds.

The mud weight of all the studied samples is given in Figure 3 which reveals that the concentration of GNP gave an insignificant effect on the mud weight of the studied samples. It could be due to the negligible dimension of its particle size. It is well known that the mud weight can be computed by dividing the sum of weights by the sum of volumes. In this case, the mud weight measurements will not be influenced since the added nanoparticle has a negligible weight. From Figure 3, the density of the control sample is 9.83 ppg which is a common mud weight used during the initial stage of a drilling operation. The ability of the mud to carry drilled cuttings is a key feature of mud density evaluation, as the suspending mud has a buoyancy influence on the cuttings. The density increase is less than 0.2 ppg with the addition of graphene nanoplatelets at concentrations of 0.3–1.5 g, which is considered a very minimal increase. The average mud weight was found 9.84 with a standard deviation of 0.00756. Similar trends have also been observed in the literature [21].

### 3.4. Rheological Properties

**Figure 4:**
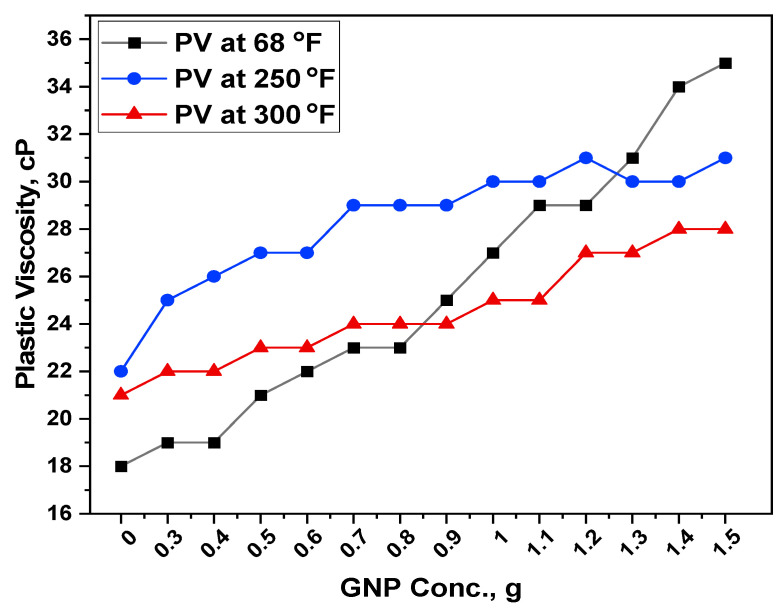
Response of GNP-Starch on the plastic viscosity (PV) of the formulated muds.

**Figure 5:**
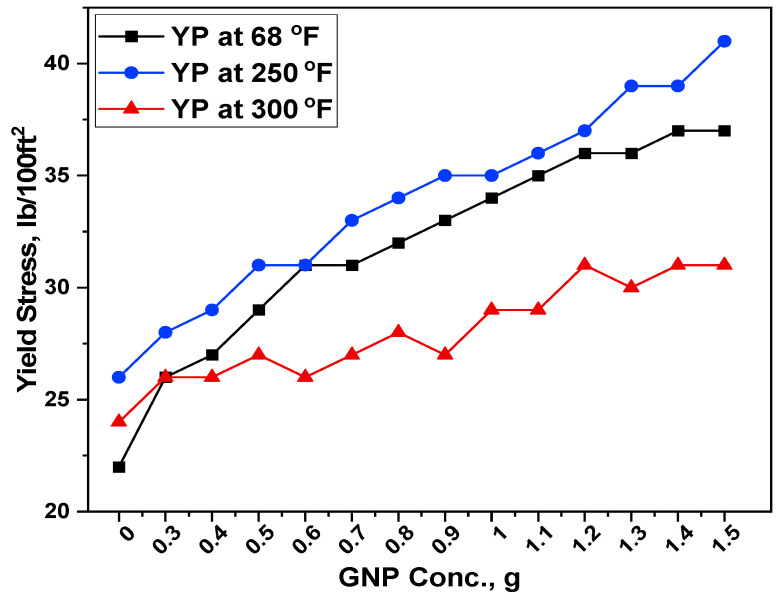
Response of GNP-Starch on the YP of the formulated muds.

The addition of GNPs improved the rheological performance of WBM when utilized with starch particles. Figure 4 and Figure 5 show the rheological properties of the formulated drilling muds, including plastic viscosity and yield stress. 

#### 3.4.1. Plastic Viscosity

The introduction of GNP enhanced PV because it caused flow resistance owing to friction between the GNPs, other mud additives, and the mud’s liquid phase. The PV has also increased as the concentration of solids in the drilling mud has increased. It is believed that GNP has a higher PV because of friction between the nanoplatelets, micro additives, and the mud’s liquid medium.

Figure 4 illustrates the PV for a water-based mud containing GNP and starch before ageing (68 °F). The PV for base mud was recorded as 18 cP while it was increased to 19 cP with the addition of 0.3 g of GNP. This corresponds to an increase of 5.56% in the PV. With a further increase in concentration to 0.4, 0.5, 0.6, 0.7, 0.8, 0.9, 1, 1.1, 1.2, 1.3, 1.4 and 1.5 g, the percent increase in the PV was recorded as 5.6%, 16.7%, 22.2%, 27.8%, 27.8%, 38.9%, 50%, 61.1%, 61.1%, 72.2%, 88.9% and 94.4% respectively. This improvement in plastic viscosity is most likely related to the degree of GNP dispersion, which influences the mud’s rheological behavior. Lower plastic viscosity in non-payzone section emerges issues like poor cuttings transport. Thus, sufficient PV is required to overcome the cuttings settling problems.

In Figure 4, the influence of temperature on PV could be observed. The temperature showed an inverse relationship with plastic viscosity of mud blends. It is due to the fact that the cohesion and attraction forces between molecules reduces as temperature rises. Internal energy is gained by the molecules, resulting in random particle movement. As a result, the drilling fluid flows more easily.

When the temperature of the testing mud samples was increased to 250 °F, the plastic viscosity of the base mud was increased to 22 cP which could be due to the increase in the viscosity of the entire mud system because of the starch granule’s rupture. When compared with the base mud, the percent increase in PV was recorded as 13.64% and 40.91% for 0.3 and 1.5 g GNP concentration.

With a further increase in the hot rolling temperature, the PV of the base mud was reduced to 21 cP. This is due to the segregation of bentonite particles because of longer exposure to high temperatures of 300 °F. However, with the addition of 0.5 g GNP, the PV improved significantly to 22 cP. This could be due to the fact that starch molecules also assist the GNP particles and keep them in a suspended form and maintain the viscosity of the whole system. When heated, starch molecules become water soluble. This is due to the starch swelling and rupturing because of the heat and water. The semi-crystalline structure of the granule is destroyed, and smaller amylose molecules begin to leach out, establishing a network that traps water and increases the viscosity of the mixture. This is known as starch gelatinization. With the addition of more and more GNP, the viscosity partially increased and finally 28.6% increase was observed with the addition of 1.5 g of GNP. The PV of the drilling fluid is strongly dependent on the surface area of the particles in the fluid. Owing to the large surface to volume ratio of GNP, the plastic viscosity also enhanced. Although the viscosity values were not much higher as compared to the other studied temperatures, but all the PV values were in API recommended range. Because the current range of 300 °F is supposed to transport the cuttings and keep the solids in suspension while drilling is ceased, this implies that the mud may flow more easily, and the drilling operation will be considerably faster. As the higher the PV, the higher the resistance in the mud, and hence the more power required to pump the mud [39].

It is obvious that the effect of GNPs solely as an additive in mud on improving rheological properties is negligible. But when GNPs was added in the presence of starch, it performed as a crosslinking agent and formed a stronger and more interconnected network structure that improved the rheological parameters considerably. Moreover, the GNP particles further supported the mud in terms of its thermal stability. Another reason for this increase is the presence of water molecules in a heated environment that results in the hydration of the starch granules [12]. The minimum friction between the GNP, other additives, and the fluid was also considered to be the cause of the improved rheological performance.

#### 3.4.2. Yield Point

Drilling mud’s yield point (YP) refers to its capacity to carry drilled cuttings from the subsurface to the surface. Drilling mud with a high YP value may deliver drilled cuttings to the surface more effectively than drilling mud with a low YP value. On the other hand, the high YP of drilling muds demands a massive proportion of pumping power, that results in significant operational costs. Figure 5 demonstrates the response of the YP of selected drilling mud formulations measured at room and high temperature conditions. The base mud showed the YP values as 22, 26 and 24 (lb/100ft^2^) at 68 °F, 250 °F and 300 °F, respectively. With the addition of GNP, the yield stress was increased to 26, 28 and 26 (lb/100ft^2^) for the selected temperatures. This corresponds to percent increases of 18.18%, 7.69% and 8.33%. The addition of GNP to the base mud enhanced the mud properties at 68 °F and 250 °F, however the effect was less prominent when the sample was exposed to 300 °F. In all the temperatures, it was observed that the yield stress values were in the API recommended range.

The presence of well-dispersed GNP might result in a more uniform distribution of other additives within the drilling fluid, giving polymers improved hydration and thereby boosting the base fluid’s cuttings transport capacity.

Overall, the effect of starch and GNP on rheological properties revealed that adding GNP to the base mud enhanced rheological parameters. On the other hand, increases in temperature showed a detrimental impact on the rheological parameters. The mud samples’ rheological properties were reduced due to the disintegration of the three-dimensional card house structure.

To evaluate drilling mud efficiency during well cleaning operations, the yield point and plastic viscosity (YP/PV) relationship is considered an important parameter. A combination of a lower plastic viscosity and a high yield point demonstrates the optimal performance of the mud. It describes the viscosity influence on the well-cleaning operations that is a characteristic of the shear rate for the Bingham fluids. Furthermore, the YP/PV ratio reflects the flow pattern of drilling muds, and a greater YP/PV ratio is needed to increase the pseudo-plastic characteristics of drilling muds. Furthermore, it improves cutting transportation efficiency, resulting in improved pump performance and an increase in the optimal drilling rate.

**Figure 6:**
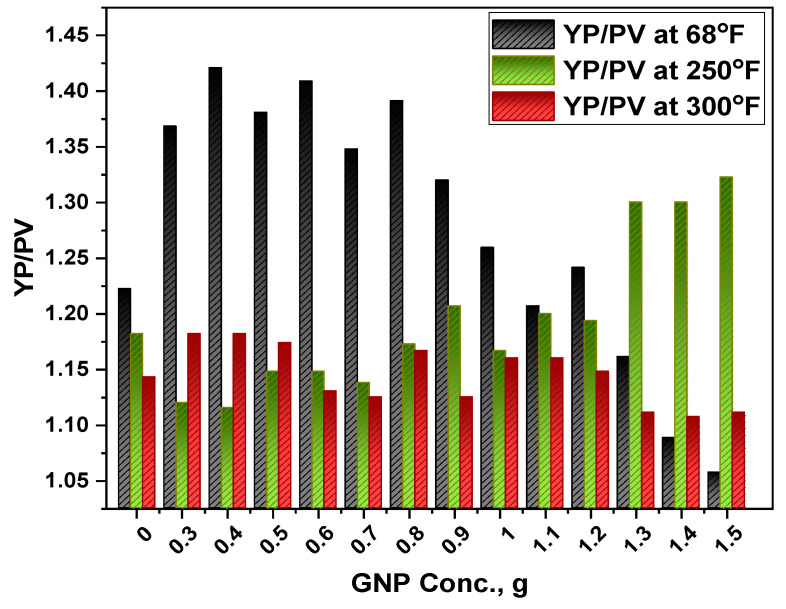
Response of GNP-Starch on the yield point/plastic viscosity (YP/PV).

Figure 6 illustrates that the base mud’s YP/PV ratio was reduced by 3.27 and 6.56% following the hot roll at 250 °F and 300 °F respectively. The mud showed the highest YP/PV value of 1.42 with GNP concentration of 0.4 g at 68 °F. After the mentioned concentration, the value was reduced and showed a minimum value at 1.5 g. this shows that the cuttings could be transported effectively to the surface at this concentration. With an increase in the hot roll temperature to 250 °F, the 1.5 g GNP loading showed better hole cleaning efficiency. It could be due to the increase in yield stress which showed better performance. Similarly, at 300 °F, the 0.4 g GNP loading showed an optimal value for YP/PV. As a result, adding GNP to the base mud increased the efficiency of the well-cleaning after the hot roll process. When compared to other nanoparticles tested by many researchers, the effectiveness of GNP was shown to be better in this study. Finally, the findings showed that adding GNP to drilling mud improves and maintains its rheological parameters in high-temperature environments. The statistical data showed a mean of 1.3, 1.2 and 1.1 for 68 °F, 250 °F and 300 °F respectively. Besides, he standard deviation was calculated as 0.12, 0.07 and 0.03 for the studied temperature range. 

### 3.5. Filtration Properties

**Figure 7:**
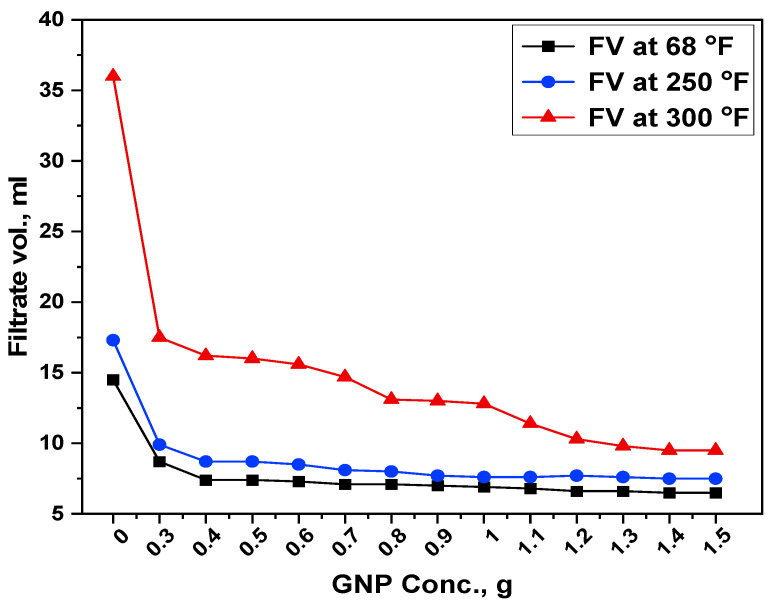
Filtrate volume of formulated mud at ambient and high temperatures.

**Figure 8:**
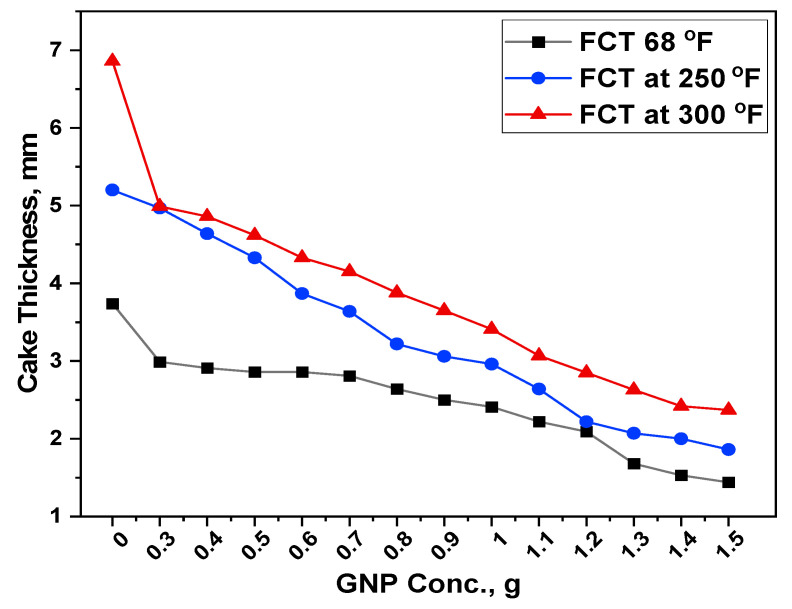
Filtercake thickness of the formulated muds at ambient and high temperatures.

When developing a drilling mud system, it is particularly important to formulate a drilling mud with a minimal filtrate loss. Higher fluid loss in a drilling operation may result in the infiltration of drilling muds, resulting in borehole instability, which may limit reservoir potential and productivity. On the other hand, GNP seems to have potential as a fluid loss reducing agent for water-based mud. Experiments have shown that combining GNP and starch reduces filtration loss in the formation. The greater effect of starch-GNP was observed in the filtration capabilities of the formulated muds. Figure 7 and Figure 8 represent the filtrate volume and filtercake thickness reduction both at room as well as at high temperature and high-pressure conditions (250 °F and 300 °F) for different blends.

#### 3.5.1. Filtrate Volume

Figure 7 illustrates the performance of water-based mud in the presence of GNP on the filtrate volume. The filtration tests were conducted at 500 psi, which is within an acceptable range of hydrostatic pressure (200–500 psi). In the base fluid without GNP, the obtained filtrate loss was 14.5 mL at room temperature. Filtrate loss was found to be reduced when GNP was added to the base mud at concentrations of 0.3, 0.4, 0.5, 0.6, 0.7, 0.8, 0.9, 1, 1.1, 1.2, 1.3, 1.4, and 1.5 g. The concentration showed a filtrate volume of 8.7, 7.4, 7.4, 7.3, 7.1, 7.1, 7, 6.9, 6.8, 6.6, 6.6, 6.5 and 6.5 mL. It can be seen that the addition of graphene nanoplatelets has effectively reduced the filtrate loss up to 55.17% at room temperature with a GNP concentration of 1.5 g. This is attributed to the fact that the added material has been completely emulsified in the mud samples, thereby forming a stable mud suspension. 

When the same samples were exposed to high pressure high temperature conditions (250 °F and 500 psi), the filtrate volume was 17.3 mL for API filtration, while the GNP addition reduced the volume in the order of 9.9, 8.7, 8.7, 8.5, 8.1, 8, 7.7, 7.6, 7.6, 7.7, 7.6, 7.5 and 7.5 mL for the selected GNP concentration. The lowest filtrate volume due to the GNP was recorded as 7.5 mL, which shows its effectiveness as a fluid loss reducer in the studied conditions. With a further increase in the temperature to 300 °F and keeping the same pressure, it can be seen that the base fluid resulted in filtrate volume of 36 mL, which corresponds to 51.4% increase when compared with the ambient temperatures. When 0.3 g GNP was added to the base mud, it resulted in a decrease of 55% at the same conditions. In addition, the 1.5 g GNP yielded a 9.5 mL fluid loss volume corresponding to 73.6% decrease in the collected volume. 

#### 3.5.2. Filtercake Thickness

While designing a mud formulation, it is very important to ensure the generated filtercake from the drilling mud is thin, smooth, and impermeable to avoid the associated drilling problems such as drill bit sticking and formation damage. Figure 8 illustrates the effect of GNP on the filtercake thickness in both ambient and high temperature conditions. The findings revealed that the control sample showed a cake thickness of 3.74 mm. When 0.3 g of GNP was added to the base mud, the cake thickness was reduced to 2.99 mm which is a decrease of about 20%. With the addition of 1.5 g, it showed a minimum filtercake thickness of 1.44 mm, which corresponds to 61.5% decrease in its thickness.

Similarly, increasing the temperature of the base mud to 250 °F resulted in a higher thickness of 5.2 mm, indicating that the proper network had not been developed to stop the fluid flowing through the filter, resulting in the formation of a permeable cake that allowed the fluid to pass through it. Because of the high temperature, the bentonite particles disintegrated and lost their functions, whereas the starch granules retained some of the particles due to their gelling ability at such a high temperature. When the temperature of the cell was raised to 300 °F at the same pressure, the control sample showed a filtercake thickness of 6.86 mm. This thickness is considered much greater and inadequate, allowing the liquid part of the mud to filter through the medium. The addition of 0.3 g of GNP into the sample resulted in a cake thickness of 4.99 mm, which is much better compared to the base mud. This reduction in thickness corresponds to 27.3%. Because the smallest size of GNP particles plugs the micro channels between the mud particles, resulting in blockage of the channels. It is significant that each component in the mud system has a specific role. For instance, the starch granules at higher temperatures increased the mud viscosity and kept the GNP in a dispersed form, whereas the GNP helped to control the fluid loss by plugging the micro-fractures in the filtercake.

From Figure 7 and Figure 8, it seems that addition of the GNP decreases the fluid loss volume and cake thickness even at a lower concentration. The is due to the repulsive forces between the GNP and the bentonite that caused the filtrate volume to decline, preventing particle aggregation while keeping the fluid in a well-distributed system at the same time. The scattered non-coagulated structure of particles in the filtercake is compacted as a result of the pressure applied in the filtration process, reducing the porosity and permeability of the cake, resulting in a decrease in filtration loss volume.

Overall, the filtration behavior of GNP was superior to that of base mud in both ambient and HPHT conditions. This improvement in filtration parameters is due to its better thermal stability, well-dispersibility and creation of a complex network structure with other mud additives. Despite that, both starch and GNP enhanced the filtration properties, but GNP performed better by plugging the micro-fractures and reducing the filtrate volume. The trend of both API and HPHT filtration showed the same decreasing trend with the addition of GNP to the base mud. Similar trends have been found by other researchers [40,41,42]. These findings demonstrate that GNP might preserve polymer characteristics in order to limit filtrate loss and, as a consequence, enhance drilling mud efficiency after the hot roll process.

### 3.6. Comparative Analysis

To validate the accuracy of the current work, the experimental results were compared with the latest research focused on nanoparticles utilization in WBMs. Table 4 summarizes the results obtained in this work with other previously published papers. The rheological properties in terms of PV and YP were found in API recommended range [43]. Both plastic viscosity and yield point values were affected with the increase in the hot roll temperature but still the values were found in the acceptable range. Additionally, the fluid loss volume and filtercake thickness also showed significant decrease with the addition of GNPs. It was observed from the current study that GNP in addition with starch significantly improved the bentonite based mud properties. 

### 3.7. Filtercake Texture and Morphology

The texture of the mud filtercake is one of the elements to consider when assessing the drilling mud’s performance. The type of additive used in the mud formulation controls the features of the mud filter cake, such as smoothness or coarseness, as well as the number of spaces and cracks. From the experimental work, it was noticed that the mud filtercake developed by the GNP is comparatively smooth textured as compared to the base mud at ambient temperature conditions. Likewise, the filtercake formed after HPHT tests displayed a coarser filtercake of the base mud as compared to the GNP based mud blends. This could be due to the disintegration of bentonite particles, resulting in a lumped structure and keeping voids between bentonite particles. With the introduction of GNP, the smoothness increased because of the covering of pore spaces between the particles. This plugging is attributed to the smallest size of GNP that penetrate into the micro-pores of the filter medium and filtercake.

**Figure 9:**
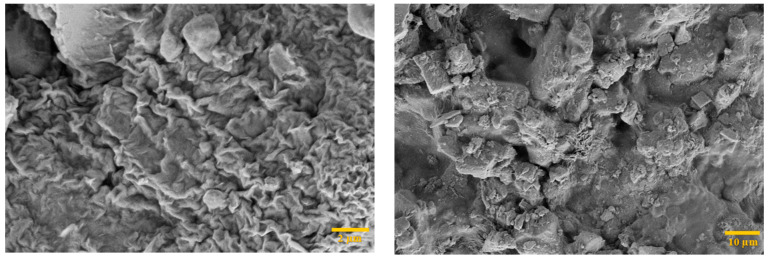
FESEM images of filtercakes of optimized samples.

Figure 9 illustrates the surface morphology of filtercake produced from the mud samples. The filtercakes had a smooth and reduced thickness and showed minimum pores for the passage of filtrate. The GNP created a strong network with starch granules that limited the fluid flow. Although the starch in addition to GNP didn’t completely stopped the filtrate flow at higher temperatures but still an acceptable amount of fluid loss volume has been obtained. 

## 4. Conclusions

The combined effect of graphene nanoplatelets and starch in bentonite-based mud has been examined using a series of lab experiments. Due to the higher thermal stability, active surface area and effective dispersion properties of GNP, these showed an improvement in the rheological properties of water-based muds. The percent increase in plastic viscosity was recorded as 94.4%, 47.6% and 33.3% for 68 °F, 250 °F and 300 °F, respectively. The yield point was also enhanced from 22–37 lb/100ft^2^, 26–41 lb/100ft^2^ and 24–31 lb/100ft^2^ at the studied range which is a suitable range for cuttings transportation. The fluid loss experiments revealed that the addition of GNP to the base mud assisted in the development of a strong and rigid structure network created by its interaction with other additives, which reduced mud sample filtration loss under both API and HPHT conditions. The percent fluid loss was dramatically reduced as 55.2%, 56.6% and 73.6% at 68 °F, 250 °F and 300 °F respectively. The filtercake thickness was also reduced upon adding the GNP to the mud samples. Graphene nanoplates showed superior filtration properties even at lower loadings. The morphological analysis showed that the GNP limited the filtrate flow by plugging the channels created between other mud agents at high temperature and pressure conditions. This study indicated that the use of GNP and tapioca starch in lab-scale experiments resulted in significant improvements in mud properties. Therefore, it is recommended to evaluate and compare these changes in mud characteristics in the field-used mud against the laboratory-prepared mud. Furthermore, the effect of different salt concentrations should be investigated in order to comprehend the combined effect of the starch and GNPs.

## Figures and Tables

**Table 1:** Composition of bentonite.

Component	Percentage %
SiO_2_	66.16
Al_2_O_3_	15.42
Na_2_O	1.93
MgO	3.46
CaO	1.23
TiO_2_	0.19
Fe_2_O_3_	5.30

**Table 2:** Water-based drilling muds formulation.

Materials	Function (s)	Dosage
Deionized (DI) water, mL	Base fluid	350
Bentonite, g	Primary viscosifier	12
Barite, g	Weighting agent	65
Potassium hydroxide (KOH), g	pH control	0.25
Polyanionic cellulose (PAC-R), g	Fluid loss control	2
Starch, g	Rheology and filtration	6
Graphene nanoplatelets, g	Rheology and filtration	0.3–1.5

**Table 3:** Drilling fluid formulations.

Additive	Formulations													
	S0	S1	S2	S3	S4	S5	S6	S7	S8	S9	S10	S11	S12	S13
DI Water, mL	350	350	350	350	350	350	350	350	350	350	350	350	350	350
Bentonite, g	12	12	12	12	12	12	12	12	12	12	12	12	12	12
Barite, g	65	65	65	65	65	65	65	65	65	65	65	65	65	65
KOH, g	0.25	0.25	0.25	0.25	0.25	0.25	0.25	0.25	0.25	0.25	0.25	0.25	0.25	0.25
PAC-R, g	2	2	2	2	2	2	2	2	2	2	2	2	2	2
Starch, g	6	6	6	6	6	6	6	6	6	6	6	6	6	6
GNPs, g	-	0.3	0.4	0.5	0.6	0.7	0.8	0.9	1.0	1.1	1.2	1.3	1.4	1.5

**Table 4:** Comparative analysis of the literature and current study.

Author	Nanoparticles Used	PV, cP	YP, lb/100ft^2^	FLV, mL	FCT, mm
Alam, et al. [44]	Iron oxide NPs	~20	~35	~6.1–7.2	-
Pakdaman, et al. [45]	SiO_2_ NPs	17–27	10.2–14.8	3.2– 20.2	~2.4
Aftab, Ismail and Ibupoto [25]	GNPs	23	29.17	5.5–14	-
Perumalsamy, et al. [46]	Esters and GNPs	5.20–12.20	4.40–18	7.40–20.60	-
Ahmed, et al. [47]	Iron oxide NPs	~22–23	~30–33	5.2–5.3	~0.95–1.15
Ali, et al. [48]	SiO_2_@ZnO@Xanthan nanocomposite	10–22	30–37	5–10.2	0.3–3.5
Keshavarz Moraveji, et al. [49]	Amorphous silica NPs	15–20	8–13	15.8–17.5	-
Hamad, et al. [50]	Silica NPs	12–20	8–19	8.3–16.5	-
Current work	GNPs	19–35	22–37	6.5–17.5	1.44–4.99

## Data Availability

The data presented in this study are available on request from the corresponding author.
